# Parental mentalization across cultures: Mind‐mindedness and parental reflective functioning in British and South Korean mothers

**DOI:** 10.1002/imhj.22151

**Published:** 2024-12-08

**Authors:** Yujin Lee, Elizabeth Meins, Fionnuala Larkin

**Affiliations:** ^1^ Department of Psychology University of York York UK; ^2^ Department of Psychology University College Cork Cork Ireland

**Keywords:** cultural differences, maternal mind‐mindedness, maternal well‐being, parental reflective functioning, parenting, **الكلمات المفتاحية**: الاختلافات الثقافية، التأمل العقلي للأمهات، الأداء الذهني الانعكاسي للوالدين ، الأبوة والأمومة، رفاهية الأمهات, 关键词:文化差异, 母亲心智, 父母反思功能, 育儿, 母亲健康, Différences culturelles, orientation mentale maternelle, fonctionnement réflexif parental, parentage, bien‐être maternel, Kulturelle Unterschiede, Mütterliche Mind‐Mindedness, Parental Reflective Functioning, Mütterliches Wohlbefinden, 文化的な差、母親のマインド・マインデッドネス、親の省察的機能、子育て、母親のウェルビーイング, diferencias culturales, actitud materna de prestar atención conscientemente, Funcionamiento con Reflexión de los Progenitores, crianza, bienestar materno

## Abstract

Differences in mind‐mindedness and parental reflective functioning (PRF) were investigated in mothers and their 6‐month‐old infants from South Korea (*N *= 66, 32 girls) and the United Kingdom (*N *= 63, 26 girls). Mind‐mindedness was assessed in terms of appropriate and non‐attuned mind‐related comments during infant–mother interaction; PRF was assessed using a questionnaire. British mothers commented more on infant desires and preferences, whereas Korean mothers commented more on cognitions and emotions, but there were no cultural differences in overall levels of mind‐mindedness. For PRF, Korean mothers reported more certainty about their infants’ mental states compared with their British counterparts, but there were no cultural differences in mothers’ reported interest in their infants’ mental states. Greater reported certainty about infants’ mental states was positively related to self‐reported parenting quality in both cultural groups, but this association was not seen for parenting quality as assessed observationally. Mind‐mindedness and PRF were unrelated in both Korean and British mothers. Results are discussed in terms of the Korean concept of mother–infant *oneness* and the multi‐dimensional nature of parental mentalization.

## ملخص

تم التحقيق في الاختلافات في التصور الذهني والأداء التأملي للوالدين لدى الأمهات وأطفالهن الرضع البالغين من العمر 6 أشهر من كوريا الجنوبية (العدد = 66، 32 فتاة) والمملكة المتحدة (العدد = 63، 26 فتاة). وتم تقييم التصور العقلي من حيث التعليقات الملائمة وغير الملائمة المتعلقة بالعقل أثناء التفاعل بين الرضيع والأم، وتم تقييم الأداء العقلي التأملي باستخدام استبيان. علقت الأمهات البريطانيات أكثر على رغبات الرضيع وتفضيلاته، بينما علقت الأمهات الكوريات أكثر على الإدراكات والعواطف، ولكن لم تكن هناك اختلافات ثقافية في المستويات العامة للتصور الذهني. بالنسبة للتركيز العقلي للرضيع، أبلغت الأمهات الكوريات عن يقين أكبر بشأن الحالات العقلية لأطفالهن الرضع مقارنة بنظيراتهن البريطانيات، ولكن لم تكن هناك فروق ثقافية في اهتمام الأمهات المبلغ عنه بالحالات العقلية لأطفالهن الرضع. ارتبط اليقين الأكبر المبلغ عنه بشأن الحالات العقلية للرضع ارتباطًا إيجابيًا بجودة التربية المبلغ عنها ذاتيًا في كلتا المجموعتين الثقافيتين، ولكن لم يظهر هذا الارتباط بالنسبة لجودة التربية كما تم تقييمها بالملاحظة. لم يكن هناك ارتباط بين اليقين الذهني وتقييم جودة التربية الذاتية لدى الأمهات الكوريات والبريطانيات. نوقشت النتائج من حيث المفهوم الكوري لوحدة الأم والرضيع والطبيعة متعددة الأبعاد للتصور الذهني للوالدين.

## 摘 要

本研究探讨了韩国(*N* = 66, 女婴32名)和英国(*N* = 63, 女婴26名)母亲及其6个月大婴儿在心智和父母反思功能(PRF)方面的差异。心智通过母婴互动期间母亲发表的与婴儿心理状态相关的恰当评论和不匹配评论进行评估;父母反思功能通过问卷调查进行评估。英国母亲更倾向于评论婴儿的愿望和偏好, 而韩国母亲则更多地评论婴儿的认知和情绪, 但在心智的总体水平上, 两组母亲之间没有文化差异。在父母反思功能方面, 与英国母亲相比, 韩国母亲对婴儿的心理状态更确定, 但两组母亲在对婴儿心理状态的兴趣报告上无文化差异。在两个文化群体中, 对婴儿心理状态的更高确定性与母亲自我报告的育儿质量呈正相关, 但这一关联未在基于观察的育儿质量评估中得到体现。在韩国和英国母亲中, 心智与父母反思功能之间均未显示相关性。研究结果结合韩国文化中“母婴合一”的概念以及父母心智化的多维性特征进行了讨论。

## French

Les différences dans l’état d'esprit (orientation mentale) et le fonctionnement réflexif parental (ici abrégé en français FRP) ont été étudiées chez des mères et leurs bébés de 6 mois en Corée du Sud (*N* = 66, 32 filles) et au Royaume‐Uni (*N* = 63, 26 filles). L’état d'esprit (orientation mentale) a été évalué en fonction de commentaires soit appropriés soit non adaptés au cours de l'interaction bébé‐mère; le FRP a été évalué avec questionnaire. Les mères britanniques ont davantage commenté les désirs et préférences des nourrissons, tandis que les mères coréennes ont plus commenté les cognitions et les émotions, mais il n'y avait aucune différence culturelle dans les niveaux généraux de l'orientation mentale. Pour ce qui concerne le FRP, les mères coréennes ont rapporté avoir plus de certitude quant à l’état mental de leur enfant, comparées aux mères britanniques, mais il n'y avait aucune différence culturelle dans l'intérêt que les mères ont manifesté à l’égard de l’état mental de leur bébé. La plus grande certitude signalée quant à l’état mental des nourrissons était liée de manière positive à la qualité du parentage auto‐rapportée dans les deux groupes culturels, mais ce lien n'a pas été trouvé pour la qualité du parentage évaluée par observation. L’état d'esprit et le FRP n’étaient pas liés chez les mères coréennes et britanniques. Les résultats sont discutés en termes du concept coréen de l'unité mère‐enfant et de la nature multidimensionnelle de la mentalisation parentale.

## German

Es wurden Unterschiede in der Mind‐Mindedness und der elterlichen Mentalisierungsfähigkeit (Parental Reflective Functioning, PRF) bei Müttern und ihren 6 Monate alten Säuglingen aus Südkorea (*N* = 66, 32 Mädchen) und dem Vereinigten Königreich (*N* = 63, 26 Mädchen) untersucht. Britische Mütter äußerten sich eher zu den Wünschen und Vorlieben des Säuglings, während koreanische Mütter sich eher zu Kognitionen und Emotionen äußerten, aber es gab keine kulturellen Unterschiede im Gesamtniveau der Mind‐Mindedness. Bei der PRF berichteten koreanische Mütter im Vergleich zu britischen mehr Gewissheit über die mentalen Zustände ihrer Säuglinge, aber es gab keine kulturellen Unterschiede im Interesse der Mütter an den mentalen Zuständen ihrer Säuglinge. Eine größere berichtete Gewissheit über die mentalen Zustände der Säuglinge stand in beiden kulturellen Gruppen in einem positiven Zusammenhang mit der selbst eingeschätzten Erziehungsqualität, aber dieser Zusammenhang wurde nicht für die beobachtete Erziehungsqualität festgestellt. Mind‐Mindedness und PRF standen sowohl bei koreanischen als auch bei britischen Müttern in keinem Zusammenhang. Die Ergebnisse werden im Hinblick auf das koreanische Konzept der Mutter‐Kind‐Einheit und die multidimensionale Natur elterlicher Mentalisierung diskutiert.

## 抄録

韓国(*N* = 66、女児32人)とイギリス(*N* = 63、女児26人)の母親と生後6ヶ月の乳児を対象に、マインド・マインデッドネスと親の省察的機能(PRF)の違いを調査した。マインド・マインデッドネスは、乳児と母親の相互作用の中で、心に関連する適切なコメントとそうでないコメントという観点から、PRFは質問紙を用いて評価した。イギリス人の母親は乳児の欲求や嗜好についてより多くコメントしたのに対し、韓国人の母親は認知や感情についてより多くコメントしたが、全体的なマインド・マインデッドネスのレベルに文化差はなかった。PRFについて、韓国人の母親はイギリス人の母親と比較して、乳児の精神状態についてより確信があると報告したが、乳児の精神状態に対する母親の関心については文化的な差はなかった。乳児の精神状態についてより確信があると報告されたことは、どちらの文化集団においても、自己報告された子育ての質と正の関連を示したが、観察的に評価された子育ての質については、この関連は見られなかった。マインド・マインデッドネスとPRFは、韓国人および英国人の母親において関連性はなかった。結果は、韓国における母親と乳児の一体性の概念と、親のメンタライゼーションの多次元的性質という観点から考察された。

## Resumen

Se investigaron las diferencias en la actitud de prestar atención conscientemente y el Funcionamiento con Reflexión de los Progenitores (PRF) en mamás y sus infantes de 6 meses de edad de Corea del Sur (*N* = 66, 32 niñas) y el Reino Unido (*N* = 63, 26 niñas). Se evaluó la actitud de prestar atención conscientemente en términos de comentarios apropiados y aquellos no a tono con relación a la mente durante la interacción infante‐mamá; PRF fue evaluado usando un cuestionario. Las mamás británicas comentaron más acerca de los deseos y preferencias del infante, mientras que las mamás surcoreanas comentaron más sobre cogniciones y emociones, pero no hubo diferencias culturales en niveles generales de la actitud de prestar atención conscientemente. Para PRF, las mamás surcoreanas reportaron más certeza acerca de los estados mentales de sus infantes comparadas con su contraparte británica, pero no hubo diferencias culturales en cuanto al reportado interés de las mamás en los estados mentales de sus infantes. La mayor reportada certeza acerca de los estados mentales de sus infantes se relacionó positivamente con la auto reportada calidad de la crianza en ambos grupos culturales, pero esta asociación no se consideró en cuanto a la calidad de la crianza como evaluada por observación. La actitud de prestar atención conscientemente y PRF no se relacionaron en ambos casos, las mamás surcoreanas y las británicas. Se discuten los resultados en términos del concepto surcoreano de unidad madre‐infante y la naturaleza multidimensional de la mentalización del progenitor.

## 1 INTRODUCTION

Parental mentalization refers to parents’ ability to understand their children's states of mind and employ this comprehension to interpret and predict their children's behavior (Meins, 1997; Sharp & Fonagy, 2008). *Mind‐mindedness* and *parental reflective functioning* (PRF) are the two parental mentalization constructs that have been most actively studied to date and are the focus of the present study. Mind‐mindedness is defined as the parent's proclivity to treat their infant as an individual with a mind of their own (Meins, 1997). The original measure assessed mind‐mindedness in terms of the extent to which the caregiver focuses on mental and emotional characteristics when invited to describe their preschooler (Meins et al., 1998). More recently, an observation‐based measure was developed to assess mind‐mindedness during infancy. This measure operationalizes mind‐mindedness in terms of the caregiver's tendency to comment on the infant's internal states in an appropriate versus non‐attuned manner (Meins et al., 2001, 2012). Appropriate mind‐related comments indicate caregivers’ accurate interpretations of the infant's internal state (e.g., stating the infant is interested in a toy car if they have played with it for a sustained period of time), whereas non‐attuned mind‐related comments index misinterpretations of the infant's thoughts and feelings (e.g., stating the infant is tired in the absence of any behavioral sign of tiredness). Mind‐mindedness is characterized as scoring highly for appropriate mind‐related comments and/or making no or few non‐attuned mind‐related comments.

PRF refers to a parent's ability to represent their child's thoughts, feelings, and beliefs, and thus hold their child's mental experiences in mind (Slade, 2005). It is often assessed from analyzing transcripts of interviews such as the Parent Development Interview (Aber et al., 1985), which deals with parenting and the parent–child relationship. A questionnaire measure of PRF has also been developed. The Parental Reflective Functioning Questionnaire (PRFQ; Luyten et al., 2017) operationalizes PRF according to three subscales: (a) *Pre‐mentalizing modes* indicates parents’ tendency not to engage with their children's mental states or to make bizarre mental state attributions, (b) *Certainty about mental states* indexes parents’ understanding of the opacity of mental states, and (c) *Interest and curiosity in mental states* assesses parents’ genuine interest in their children's thoughts and feelings.

### 1.1 Parental mentalization and parenting practices

Previous research has reported positive associations between caregivers’ appropriate mind‐related comments and their behavioral responsivity when interacting with their infants in free‐play contexts, whereas non‐attuned mind‐related comments are unrelated to responsivity (e.g., Meins et al., 2012, 2001; Shai & Meins, 2018). However, non‐attuned comments have been reported to relate to caregiver responsivity in more stressful contexts. For example, McMahon & Newey (2018) reported that mothers’ non‐attuned comments were negatively associated with their emotional availability to the infant during the Still Face Paradigm (Tronick et al., 1978).

**Key Findings**

1. Cultural differences in maternal mind‐mindedness between British and South Korean mothers were found in the contents of mind‐related comments, rather than in the proportions of such comments or the accuracy of interpreting their infants’ internal states.
2. Korean mothers scored higher in certainty about mental states than British mothers, with this certainty correlating with self‐reported, but not observed, parenting quality in both groups.
3. The study, the first to examine the relations between mind‐mindedness and parental reflective functioning across cultures, found no associations between the two, suggesting parental mentalization is multidimensional.

**Statement of Relevance**

Parental mentalization is crucial for establishing attachment security in infancy and promoting better developmental outcomes, such as self‐regulation and school readiness. Despite its significant impact on infant development, there is limited research on how parental mentalization varies across cultures and relates to different parenting practices. By examining parental mentalization through both mother–infant interactions and retrospective questionnaire measures completed by mothers, we can deepen our understanding of cultural differences in parental mentalization.

With respect to the different dimensions of PRF assessed by the PRFQ, Moreira and Fonseca (2023) reported that pre‐mentalizing modes scores were positively correlated with self‐reported authoritarian and permissive parenting styles and negatively correlated with reported authoritative style, with a large effect size for the correlation with authoritarian style. In contrast, there were large effects for the positive associations between self‐reported authoritative parenting style and scores for both the interest and curiosity in mental states and certainty about mental states subscales. These two PRFQ subscales were also negatively correlated with reported authoritarian style but were unrelated to reported permissive style. These findings suggest that the pre‐mentalizing dimension of PRF is associated with less optimal parenting, whereas interest and curiosity in mental states and certainty about mental states are associated with more optimal parenting. Interestingly, in their development and preliminary validation of the PRFQ, Luyten et al. (2017) suggested that optimal PRF may be characterized in terms of lower pre‐mentalizing modes, higher interest and curiosity in mental states, and mid‐range certainty about mental states. However, subsequent findings indicate that the certainty about mental states subscale scores may be linearly related to positive aspects of parenting (e.g., Moreira & Fonseca, 2023).

This positive association between more optimal parenting and certainty might be seen especially in relation to self‐report measures of parenting. For example, caregivers who report certainty about their children's mental states are also likely to report feeling confident in their more general parenting abilities. Self‐reported certainty about the child's mental states and ability to parent effectively may thus both depend on the same underlying representation of oneself as a competent parent who is in control of parenting the child. However, in the absence of independent data on the quality of parenting, it is impossible to establish whether individuals who report being effective parents engage in more optimal parenting behaviors when actually interacting with the child. Including both self‐report and observational measures of parenting quality is therefore essential for addressing this question.

### 1.2 Cultural variation in parenting practices and parental mentalization

The relation between parental mentalization and parenting abilities is also likely to vary as a function of culturally specific desired and esteemed parenting practices. Lee et al. (2021) recently developed a Korean translation of the PRFQ. They reported that parents’ scores for certainty about their children's mental states were positively correlated with self‐reported quality of parenting, and proposed that high certainty about mental states may be normative—and deemed optimal—in Korean parents. This aligns with the results of Aival‐Naveh et al.’s (2019) systematic review of cultural differences in mentalizing and how it may be influenced by cultural value preferences and parenting characteristics.

A unique aspect of Korean parenting is its focus on *oneness*: viewing the mother–infant dyad as a single entity, rather than representing the mother and infant as separate individuals (Kim & Choi, 1994; Kim et al., 2005). Pursuing oneness is implicitly and explicitly manifested in Korean parenting customs; indeed, once a Korean woman becomes a mother, she is referred to as “[child's name's] mother” instead of her first name in social situations involving children. The construct of oneness characterizes ideal parenting—for mothers in particular—as having privileged and highly accurate insight into the infant's internal states and knowing exactly what the infant is thinking and feeling. The statements in the PRFQ that are indicative of certainty about mental states (e.g., “I always know what my child wants”) may thus be seen as socially desirable for Korean mothers, and explain the observed positive association between certainty about mental states and self‐reported parenting quality in Lee et al.’s (2021) study. However, research has not yet investigated differences in the dimensions of the PRFQ between Korean parents and those in individualistic Western cultures. Indeed, no study has yet investigated cross‐cultural differences in the PRFQ.

Similarly, few studies have addressed cultural differences in mind‐mindedness. Hughes et al. (2018) reported that Hong‐Kong Chinese mothers were less likely than their British counterparts to describe their 4‐year‐olds in a mind‐minded way. Fujita & Hughes (2020) reported that, compared with British mothers, Japanese mothers were less mind‐minded in their descriptions of their young children, and referred more to their own expectations rather than their children's characteristics. Dai et al. (2019) conducted the only cross‐cultural study of mind‐mindedness using the observational measure during infancy. They reported that mainland Chinese mothers made fewer mind‐related comments compared to Australian mothers, with Chinese mothers scoring less highly for appropriate comments and more highly for non‐attuned comments than their Australian counterparts. Moreover, whereas Australian mothers used “want” and “like” for referring to the child's desires and preferences significantly more than their Chinese counterparts, Chinese mothers more frequently used “want” in non‐attuned comments that resulted from their attempts to redirect their infants away from activities in which they were already engaged. However, given that this is the only cross‐cultural mind‐mindedness study during infancy, it is crucial to investigate whether this pattern generalizes to other Eastern and Western cultures.

The main aim of the present study was to explore differences in parental mentalization between the United Kingdom (UK) and South Korea, investigating cultural differences in the PRFQ dimensions and mind‐mindedness. Following Lee et al.’s (2021) proposal regarding the optimal nature of high certainty about their infants’ mental states in Korean mothers, we hypothesized that Korean mothers would achieve higher scores for certainty about mental states compared to their British counterparts. Cultural differences in the other aspects of PRF assessed by the PRFQ (pre‐mentalizing modes and interest and curiosity in mental states) were investigated without specific hypotheses. The present study also included both observational and self‐report measures of parenting quality to attempt to replicate Lee et al.’s finding that high reported certainty about the infant's mental states was related to more optimal parenting in Korean mothers, and explore whether any such relation applied to both self‐reported and independently assessed quality of parenting. The cross‐cultural design of our study enabled us to investigate whether any such relation was specific to Korean mothers and could therefore be explained in terms of the Korean concept of oneness.

Turning to predictions for cultural differences in mind‐mindedness, the findings discussed above indicating lower levels of mind‐mindedness in Chinese and Japanese mothers do not mean that Korean mothers will also be less mind‐minded than their Western counterparts. Korean parenting style differs not only from that typically seen in individualistic cultures, but also from typical Chinese and Japanese parenting practices. Chinese parenting values emphasize “training” (i.e., *guanjiao*), via which parents structure their children's behavior according to collectivistic values (e.g., Chao, 1994), which may explain the findings that Chinese mothers are less likely than their Western counterparts to describe their children with reference to their mental characteristics (Hughes et al., 2018), and more likely to intervene in their infants’ activities and state that they want to engage in activities that the parent is suggesting (Dai et al., 2019). Typical Japanese parenting is based on the concept of *mimamoru* (watching over from a distance) (Holloway, 2017), which may explain why these parents tended to describe their children in terms of their own expectations rather than their children's characteristics (Fujita & Hughes, 2020).

The cultural foundations of typical Chinese and Japanese parenting practices are therefore different from the Korean concept of oneness, according to which the mother and infant are viewed as a single entity. Oneness therefore goes beyond notions of emotional interdependence between mother and infant that may be held in other cultures. Due to this unique aspect of Korean parenting, we expected levels of mind‐mindedness in Korean mothers to be different from those observed in their Chinese and Japanese counterparts. Because the Korean parenting context emphasizes interconnectedness between parents and children, Korean mothers may be particularly motivated to understand their infants’ behavior in terms of its underlying internal states. We therefore hypothesized that Korean mothers would produce more appropriate mind‐related comments compared with British mothers. However, the cultural expectation for Korean mothers and infants to be “at one” may escalate the risk of mothers projecting their own thoughts and feelings onto their infants, rather than accurately interpreting the infant's internal states. Consequently, we hypothesized that Korean mothers would also be more likely than their British counterparts to make non‐attuned mind‐related comments. Previous research shows that appropriate and non‐attuned mind‐related comments are unrelated (e.g., Meins et al., 2002, 2012) and should not be considered to represent opposite poles of a unidimensional construct. Some caregivers thus score highly for both appropriate and non‐attuned mind‐related comments (Meins et al., 2012), and this pattern may be more typical in Korean mothers, given the cultural emphasis on oneness.

Moreover, we expected cultural differences to emerge in the types of internal states that mothers attributed to their infants. As Korean mothers’ beliefs and goals focus on building relational closeness and achieving oneness with their children, a sense of knowing their infants’ thoughts and feelings might be more crucial for Korean mothers than for British mothers. Therefore, we anticipated that Korean mothers may concentrate more than their British counterparts on commenting on their infants’ emotional and cognitive states. On the other hand, the fact that British mothers rear their infants in an individualistic cultural context that emphasizes independence led us to hypothesize that comments relating to the infant's individual preferences and desires will be more common in British mothers.

It is also important to consider a number of potential confounding variables in investigating cultural differences in parental mentalization. Compared with Western mothers, Korean mothers are known to have poorer mental health (e.g., O'Brien et al., 2014) and to perceive their infants as having higher negative affect (Krassner et al., 2017). These variables have also been reported to relate to parental mentalization. Mothers hospitalized for severe mental illness were found to have lower levels of mind‐mindedness than their psychologically well counterparts (Schacht et al., 2017), and recent meta‐analytic data showed a negative association between depression and PRFQ scores (Georg et al., 2023). Perceived infant temperament is associated with all subscales of the PRFQ (Álvarez et al., 2022). Finally, parenting stress has been reported to relate to lower levels of both mind‐mindedness (e.g., Larkin et al., 2021) and PRF (e.g., Nijssens et al., 2018).

### 1.3 Relations between mind‐mindedness and PRF

In exploring how culture related to mind‐mindedness and PRF, we were also able to investigate an important but neglected question: how do these two variables relate to one another? While mind‐mindedness and PRF are grouped together under the umbrella term parental mentalization, there is very little research investigating whether the two constructs are related. Establishing the nature of the relation between mind‐mindedness and PRF across cultures is important both theoretically and empirically. If the two constructs are not robustly related, then this would suggest that parental mentalization is a multidimensional, rather than unidimensional construct. Although mind‐mindedness and PRF are both characterized in terms of the caregiver's engagement with the child's internal states, they are operationalized very differently: PRF is indexed purely in terms of the mothers’ representations of the child as assessed through interviews or the PRFQ, whereas mind‐mindedness is measured from the extent to which the caregiver comments on the infant's internal states in appropriate versus non‐attuned ways during actual caregiver–infant interaction. One crucial difference between the constructs is thus that mind‐mindedness enables one to judge the accuracy of caregivers’ interpretations of their infants’ internal states in light of infant behavior, whereas PRF indexes caregivers’ more general tendency to engage with and reflect on the child's mental world. PRF may thus be necessary but not sufficient for the caregiver to be mind‐minded. In line with recent null findings for relations between PRF and mind‐mindedness (Dollberg, 2022; Krink & Ramsauer, 2021; Larkin et al., 2024), we expected negligible to small associations between mind‐mindedness and PRF in both British and Korean.

### 1.4 The present study

In summary, the present study had the following aims: (a) to investigate differences in mind‐mindedness and PRFQ dimensions between British and Korean mothers, (b) to establish how parenting practices related to the certainty about mental states subscale of the PRFQ in British and South Korean mothers, and (c) to investigate relations between mind‐mindedness and PRFQ dimensions in these two groups of mothers. In addressing these aims, we considered the potential confounding effects of parenting stress, maternal psychological wellbeing and infant temperament. Due to the Korean concept of *oneness* in relation to early mother–infant relationships, we hypothesized that (1) Korean mothers would make more appropriate and more non‐attuned comments compared with their British counterparts as they tend to seek knowledge of their infants’ thoughts and feelings to achieve a sense of oneness, with a potential risk of projecting their own mental states onto their infants, (2) British mothers would make more comments on their infants’ desires and preferences compared with Korean mothers, and (3) Korean mothers would make more comments on their infants’ cognitions and emotions compared with British mothers. We also hypothesized that (4) Korean parenting culture would lead to Korean mothers being more likely than British mothers to report high levels of certainty about their infants’ mental states, and that (5) high certainty about infant mental states would be related to more positive parenting; exploratory analyses established whether any such relation was specific to Korean mothers. Finally, we expected (6) negligible to small associations between mind‐mindedness and PRF in both British and Korean.

## 2 METHOD

### 2.1 Participants

Participants were British mothers (*n* = 63, *M* = 32.51 years, SD = 7.24 years, range 22–48 years) and their infants (37 boys, 26 girls, *M* = 6.14 months, SD = 1.55, range 3.50–9.40 months), and South Korean mothers (*n* = 66, *M* = 33.11 years, SD = 3.41 years, range 26–41 years) and their infants (34 boys, 32 girls, *M* = 7.49 months, SD = 1.15, range 4.23–10.63 months). In the British sample, 61 mothers were White, 1 was Mixed race, and 1 was Asian British; all mothers in the Korean sample were Asian. Regarding maternal education, most mothers had at least an undergraduate degree (British mothers: 92.1%, Korean mothers: 86.3%). All Korean mothers and 96.8% of British mothers were married or in a relationship with the infant's father; two British mothers reported that they were not in a relationship. Both British and Korean mothers were recruited in local communities, through the Internet, and via word of mouth. A priori power calculations to detect a medium‐size effect at 0.80 power with alpha set at .05 indicated that 51 participants per group were required for an independent samples test, 67 participants were required for a bivariate correlation, and 128 participants were required for ANCOVA with two groups and five covariates.

### 2.2 Materials and methods

Testing was conducted in a developmental research laboratory in a session that lasted approximately 20–30 min. All participants provided written informed consent, and they provided information anonymously using only ID numbers, and that they could withdraw from the study at any point. Questionnaire measures were completed in an online format. The procedure was approved by the relevant University ethics committees in the UK and the Korean ethics committee (2018‐1504‐006).

#### 2.2.1 Maternal mind‐mindedness

Maternal mind‐mindedness was measured via a 10‐min mother–infant free play. Mothers played with their infants as they would at home, and their speech was transcribed and coded following the mind‐mindedness coding manual (Meins & Fernyhough, 2015). Mind‐related comments include mothers’ comments about the infants’ (a) desires and preferences, (b) cognitions, (c) emotions, (d) epistemic states, and (e) talking on the infant's behalf. Each mind‐related comment was then classed as appropriate or non‐attuned by a trained coder. Mind‐related comments were classified as appropriate if any of the following criteria were met: (a) the coder agreed with the mother's reading of the infant's current internal state, (b) the mother's comment linked the infant's current internal state with similar events in the past or future, or (c) the comment suggested the infant would like or want a new object or activity after a lull in the interaction. Comments were classified as non‐attuned if: (a) the coder disagreed with the mother's reading of the infant's current internal state, (b) the mother's comment regarding a past or future event was not related to the infant's current internal state, (c) the mother asked what the infant wanted to do or suggested a new activity when the infant was already involved in something else, (d) the comment seemed not to be based on the infant's behavior or seemed to project the mother's internal states onto the infant, or (e) the referent of the comment was not clear. To control for verbosity, appropriate and non‐attuned mind‐related comments were calculated as a proportion of the total number of comments made during the play session.

For coding mind‐mindedness in the Korean sample, the original English manual was translated into Korean by a bilingual researcher, and the validity of the translated coding system was carefully considered with two other bilingual Korean/English speakers, one of whom was a developmental clinical psychologist and the other a developmental practitioner. As the main coder of the Korean sample (the first author) was the same as the main coder of the British sample, the consistency of coding between the two cultural samples was maintained. A randomly selected 20% of the free‐play sessions for each cultural group was coded by a second trained coder, who was a native speaker of English or Korean, and who was blind to all other measures. Inter‐rater reliability was UK *κ* = .72, and KOR *κ* = .86. Disagreements were resolved by discussion.

In addition, the mothers’ mind‐related comments were classified into categories regarding the content of the comments to further investigate differences in mothers’ mind‐related comments across cultures. The mind‐related comments were coded as one of the following exhaustive and exclusive categories described in Meins and Fernyhough's (2015) coding manual: (a) desire and preference (e.g., “Do you like the ball?”), (b) cognitions (e.g., “Do you know what it is?”), (c) intention (e.g., “Are you trying to put that one in?”), (d) emotions (e.g., “Are you happy?”), (e) epistemic states (e.g., “Are you playing games with me?”), (f) talking on the infant's behalf (any utterance that is obviously meant to be said or thought by the infant, e.g., “I don't like it, mum”), and (g) physical statement (e.g., “Are you tired?”). Note that terms in the final category can only be non‐attuned if they are categorized as mind‐related (Meins & Fernyhough, 2015). Scores for each category were calculated separately for appropriate and non‐attuned mind‐related comments and expressed as a proportion of the total number of appropriate or non‐attuned comments. There was perfect agreement between first and second trained coders with regard to the mental state categories.

#### 2.2.2 Parental reflective functioning

The PRFQ (Luyten et al., 2017) was completed to assess parents’ PRF. The PRFQ is an 18‐item questionnaire using a 7‐point Likert scale (1: strongly disagree to 7: strongly agree), designed to assess multidimensional traits of PRF with three subscales: (a) *Pre‐mentalizing modes* (6 items), (b) *Certainty about mental states* (6 items), and (c) *Interest and curiosity in mental states* (6 items). The subscale of *Pre‐mentalizing modes* measures parents’ inability to enter their children's mental states and their tendency to make malevolent attributions (e.g., “When my child is fussy, he or she does that just to annoy me”). A high score on this subscale indicates parents’ non‐mentalizing stance. The *Certainty about mental states* subscale captures parents’ tendency to be highly certain about their children's mental states (e.g., “I always know what my child wants”). A high score on this subscale reflects lack of awareness of the opacity of mental states, while an extremely low score reflects difficulty in having confidence about the child's subjective world. Lastly, the *Interest and curiosity in mental states* subscale measures parents’ genuine interest and curiosity in their children's subjective world (e.g., “I like to think about the reasons behind the way my child behaves and feels”). A high score on this subscale indicates parents’ high interest in their children's mental states. In total, the PRFQ gives each mother three separate average scores for the three subscales, ranging from 1 to 7. The British mothers completed the PRFQ in English; the Korean mothers completed the Korean translation of the PRFQ (Lee et al., 2021).

Acceptable internal reliability has been reported for the three scales in the English version of the PRFQ (Anis et al., 2020), but a recent study reported low internal reliability for the pre‐mentalizing subscale (Arkle et al., 2023). In the Korean version of the PRFQ, Lee et al. (2021) reported acceptable internal reliability for the certainty about mental states and curiosity about mental states subscales, but low internal reliability for the pre‐mentalizing subscale. Internal reliability for the present study was as follows: *Pre‐mentalizing Modes*, *α*_UK_ = .72, and *α*_KOR_ = .40; *Certainty about mental states*, *α*_UK_ = .64, and *α*_KOR_ = .83; *Interest and curiosity in mental states*, *α*_UK_ = .76, and *α*_KOR_ = .71. Due to the low reliability of the Korean *Pre‐mentalizing modes* subscale, results for this subscale were not included in the statistical analyses.

#### 2.2.3 Parenting style

The Revised Parents as Social Context Questionnaire (R‐PSCQ; Egeli et al., 2015; Skinner et al., 2005) was used to assess multiple aspects of parenting style. It consists of 30 items rated on a 4‐point Likert scale (1: not at all true to 4: very true) to index the following dimensions of parenting: (a) warmth, (b) rejection, (c) structure, (d) chaos, (e) autonomy support, and (f) coercion. Each subscale's score ranges between 5 and 20, and a higher score indicates a greater level of the corresponding parenting dimension. Egeli et al. (2015) reported acceptable internal reliability for all subscales of the R‐PSCQ. Korean parents completed the adapted Korean version of the PSCQ (Jeong & Shin, 2011). Internal reliability was as follows: warmth *α*_UK_ = .66, and *α*_KOR_ = .79; rejection *α*_UK_ = .67, and *α*_KOR_ = .68; structure *α*_UK_ = .72, and *α*_KOR_ = .70; chaos *α*_UK_ = .78, and *α*_KOR_ = .61; autonomy support *α*_UK_ = .64, and *α*_KOR_ = .60; and coercion *α*_UK_ = .75, and *α*_KOR_ = .77. Following Skinner et al. (2005), composite scores were calculated to represent positive parenting style (the sum of scores for the warm, structured, and autonomy‐supportive subscales), and negative parenting style (the sum of scores for the rejective, chaotic, and coercive subscales). The positive and negative parenting style composites had acceptable internal reliability (positive parenting, *α*_UK_ = .80, *α*_KOR_ = .81; negative parenting, *α*_UK_ = .84, *α*_KOR_ = .79).

#### 2.2.4 Maternal intrusiveness

The free‐play sessions from which mind‐mindedness was coded were also used to assess maternal intrusiveness using Miller & Sameroff's (1998) coding manual. Given that the original coding system was for 3‐min interactions, we divided the 10‐min session into three 3‐min 20‐s epochs. The total score was averaged over the three epochs. The level of intrusiveness in each epoch was coded by the degree to which a mother handled her infant roughly on scales of 0 (no intrusiveness) to 3 (predominant or high intrusiveness). A randomly selected 20% of the free‐play sessions were double coded by a second trained coder, who was blind to other measures. Inter‐rater reliability was ICC_UK _= .92, and ICC_KOR_ = .92.

#### 2.2.5 Maternal anxiety and depression

The Hospital Anxiety and Depression Scale (HADS; Zigmond & Snaith, 1983) is a 14‐item questionnaire with a 4‐point Likert scale ranging from 0 to 3. It has two subscales: anxiety (HADS‐A), and depressive symptoms (HADS‐D). Each of the two subscales has a range between 0 and 21, and the total score range is 0 to 42. A higher score indicates a greater level of anxiety/depressive symptoms. The Korean Hospital Anxiety and Depression Scale (K‐HADS; Oh et al., 1999) was used for Korean parents. Oh et al. (1999) reported acceptable internal reliability for all subscales of the K‐HADS. Composite scores for the two subscales were used in the analyses. Internal reliability was as follows: *α*_UK_ = .82, and *α*_KOR_ = .87.

#### 2.2.6 Parenting stress

The Parenting Stress Index‐Short Form (PSI‐SF; Abidin, 1990), is a 36‐item questionnaire with a 5‐point Likert scale and consists of three subscales: parental distress, parent–child dysfunctional interaction, and difficult child. Each subscale's score ranges from 12 to 60. For Korean mothers, the Korean Parenting Stress Index‐Short Form (K‐PSI‐SF; Lee et al., 2008) was used. Lee et al. (2008) reported acceptable internal reliability for all subscales of the Korean version of the PSI‐SF. Due to the different direction of scales of the K‐PSI‐SF (1: strongly disagree to 5: strongly agree) from the English version, the scores of the English PSI‐SF were reversed, and thus a low raw score indicated a low level of stress related to parenting. Internal reliability was as follows: parenting distress, *α*_UK_ = .84, and *α*_KOR_ = .81; parent–child dysfunctional interaction, *α*_UK_ = .88, and *α*_KOR_ = .83; difficult child, *α*_UK_ = .87, and *α*_KOR_ = .88.

Scores for the parenting distress and parent–child dysfunctional interaction scales were used as measures of parenting stress, and scores for the difficult child scale were used to index perceived infant temperament.

#### 2.2.7 Observed infant temperament

The car seat task from the Infant Laboratory Temperament Assessment Battery (Lab‐TAB; Goldsmith & Rothbart, 1996) was used for measuring observed infant temperament. It was designed to elicit mild anger responses from infants by restraining them for 30 s in a car seat, secured to a chair in the laboratory; a video camera was positioned to record the infant's face and body movements. This task has good predictive and concurrent validity for assessing infant temperament when used in isolation from the whole Lab‐TAB battery (Hay et al., 2014; Larkin et al., 2019). In order to score the episode, the 30 s were divided into six 5‐s epochs to code intensity of (a) facial anger, (b) facial sadness, (c) distress vocalizations, and (d) struggle. Higher scores indicate greater negative affect, distress, and struggle, suggestive of a more difficult temperament. A randomly selected 20% of the Lab‐TAB sessions was double coded by a second trained coder, who was blind to other measures. Inter‐rater reliability for the ratings were: facial anger, ICC_UK_ = .97, and ICC_KOR_ = .77; facial sadness, ICC_UK_ = .89, and ICC_KOR_ = .93; distress vocalization, ICC_UK_ = .97, and ICC_KOR_ = .94; struggle, ICC_UK_ = .80, and ICC_KOR_ = .68. There was good reliability for a composite measure of the four ratings, *α*_UK_ = .81, and *α*_KOR_ = .86. The composite measure was used in the analyses.

### 2.3 Analysis plan

Cultural differences in the various parental mentalization constructs were investigated using ANCOVA or MANCOVA, with nationality added as a fixed variable and HADS scores, the PSI‐SF subscale scores (parenting distress, parent–child dysfunctional interaction, and difficult child), and Lab‐TAB scores added a priori as covariates. Additional covariates were added based on the results of preliminary analyses to investigate demographic differences between the British and Korean samples. Correlational analyses were run separately on data from the British and Korean samples to investigate relations between (a) PRFQ certainty about mental states and parenting practices, and (b) mind‐mindedness and the PRFQ dimensions. Predicted non‐significant findings were confirmed using Bayesian analyses.

### 2.4 Data availability

The data file is available via the Open Science Framework (https://osf.io/mpn95/). The study was not preregistered.

## 3 RESULTS

### 3.1 Descriptive statistics and preliminary analyses

Two British mothers did not answer the age question, and another British mother's HADS and PSI‐SF responses could not be used due to a technical problem. The mother with missing HADS and PSI‐SF data was excluded from the main analyses on cultural differences in parental mentalization reported below. Infant gender was unrelated to the mind‐mindedness and PRFQ variables in both the UK (*t*s < 1.45, *p*s > .152) and Korean (*t*s < 1.59, *p*s > .123) samples. Korean infants were older than British infants, *t*(127) = 5.63, *p* < .001, and British mothers were more highly educated than Korean mothers, *t*(127) = 3.05, *p* = .003, but the groups did not differ on maternal age, *t*(125) = 0.69, *p* = .493. Infant age and maternal education were therefore included as additional covariates in the cultural differences analyses reported below. With regard to the separate categories of mind‐related comments, only two mothers mentioned epistemic states and only eight mothers talked on the infant's behalf. These categories are therefore not used in the analyses below that involve the separate categories of mind‐related comments.

Table 1 shows the parenting style, mental health, and infant temperament data as a function of nationality. As shown in Table 1, British mothers showed more positive parenting than did their Korean counterparts for both the self‐report and observational measures. In terms of psychological well‐being, Korean mothers scored more highly than British mothers for anxiety/depression and parenting distress. However, there were no significant differences between British and Korean mothers for reported or observed infant temperament and parent–child dysfunctional interaction (see Table 1).

**TABLE 1:** Descriptive statistics and cultural differences.

	United Kingdom	South Korea	Cultural comparison	
	*M* (SD)	*M* (SD)		*p*
HADS	11.05 (5.12)	13.42 (6.50)	*t*(126) = 2.29	.024
PSI‐PD	27.32 (7.34)	32.52 (8.25)	*t*(126) = 3.75	<.001
PSI‐CDI	19.16 (5.58)	18.71 (5.81)	*t*(126) = 0.45	.657
PSI‐DC	23.27 (7.20)	23.24 (7.78)	*t*(126) = 0.02	.981
Positive parenting	53.03 (3.70)	49.85 (5.32)	*t*(127) = 3.93	<.001
Negative parenting	25.25 (5.78)	26.58 (5.96)	*t*(127) = 1.28	.204
Observed intrusiveness	0.83 (0.59)	1.28 (0.72)	*t*(127) = 3.90	<.001
Observed infant temperament	2.93 (2.01)	3.56 (2.61)	*t*(127) = 1.54	.125

HADS, The Hospital Anxiety and Depression Scale; PSI‐PD, Parenting Stress Index‐Short Form Parenting distress subscale; PSI‐CDI, Parenting Stress Index‐Short Form parent–child dysfunctional interaction subscale; PSI‐DC, Parenting Stress Index‐Short Form Difficult child subscale; SD, standard deviation.

Table 2 shows the correlations between the covariate and parental mentalization variables in the UK and Korean samples.

**TABLE 2:** Correlations between covariate variables, mind‐mindedness, and the PRFQ dimensions.

	Maternal mind‐mindedness				PRFQ			
	AMRC		NMRC		CMS		IC	
	United Kingdom	South Korea	United Kingdom	South Korea	United Kingdom	South Korea	United Kingdom	South Korea
Infant age	−.20	−.01	−.32^*^	−.11	.13	.20	−.18	.17
Maternal age	−.04	.13	.01	−.14	−.10	−.12	.25	.03
Mothers’ education	.14	.14	.16	.35^**^	−.10	.34^**^	.26^*^	.27^*^
HADS	−.13	−.12	.12	−.06	−.23	−.33^**^	.11	−.04
PSI‐PD	−.14	−.07	−.11	−.23	−.30^*^	−.46^***^	.00	−.14
PSI‐P‐CDI	−.01	−.23	−.18	−.13	−.15	−.49^***^	−.15	−.34^**^
PSI‐DC	.00	−.09	−.29^*^	−.11	−.25	−.27^*^	−.21	−.25^*^
Observed temperament	.15	.24	.03	.17	.01	.10	.21	−.01
Positive parenting	−.06	.08	−.05	.11	.41^***^	.41^***^	.31^*^	.39^**^
Negative parenting	.01	.01	−.05	−.13	−.39^**^	.46^***^	−.24	−.36^**^
Intrusiveness	−.08	−.22	.11	.19	−.19	−.13	−.21	.01

Positive parenting = sum of self‐reported warm, structured, and autonomy‐supportive behaviors; Negative parenting = sum of self‐reported rejective, chaotic, and coercive behaviors; Intrusiveness = observed maternal intrusiveness. AMRC, Appropriate Mind‐Related Comments; NMRC, Non‐attuned Mind‐Related Comments; PRFQ, Parental Reflective Functioning Questionnaire; CMS, Certainty about Mental States; IC, Interest and Curiosity in Mental States; HADS, The Hospital Anxiety and Depression Scale; PSI‐PD, Parenting Stess Index‐Short Form Parenting Distess subscale; PSI‐CDI, Parenting Stress Index‐Short Form Parent–child dysfunctional interaction subscale; PSI‐DC, Parenting Stress Index‐Short Form Difficult Child subscale. *p < .05, **p < .01, ***p < .001.

### 3.2 Differences in mind‐mindedness and PRFQ dimensions between British and Korean mothers

Table 3 shows the mind‐mindedness data for the UK and Korean samples. Cultural differences in appropriate and non‐attuned mind‐related comments were investigated using ANCOVA, with nationality added as a fixed variable and infant age, maternal education, HADS scores, the PSI‐SF subscale scores (parenting distress, parent–child dysfunctional interaction, difficult child), and Lab‐TAB scores added as covariates. Contrary to our first hypothesis, there was no main effect of nationality for appropriate mind‐related comments, *F*(8, 119) = .07, *p* = .790, η^2^ = .0002, or for non‐attuned mind‐related comments, *F*(8, 119) = 0.19, *p* = .668, *η* ^2^ = .0005.

**TABLE 3:** Descriptive statistics for parental mentalization variables as a function of nationality.

		United Kingdom	South Korea
	Mind‐related comments	Mean (SD)	Mean (SD)
Total verbosity		175.24 (48.59)	178.42 (58.28)
AMRC (proportion)	Total AMRC	.06 (.03)	.06 (.04)
	Desire and preferences	.74 (.22)	.58 (.29)
	Cognition	.13 (.17)	.21 (.23)
	Intention	.04 (.09)	.01 (.05)
	Emotion	.07 (.13)	.15 (.22)
NMRC (proportion)	Total NMRC	.02 (.02)	.01 (.01)
	Desire and preferences	.68 (.40)	.37 (.41)
	Cognition	.06 (.17)	.08 (.21)
	Intention	.01 (.06)	.02 (.11)
	Emotion	.03 (.14)	.08 (.17)
	Physical statement	.03 (.10)	.15 (.29)
PRFQ	CMS	3.94 (.89)	4.88 (.88)
	IC	6.12 (.61)	6.00 (.66)

AMRC, Appropriate Mind‐Related Comments; NMRC, Non‐attuned Mind‐Related Comments; PRFQ, Parental Reflective Functioning Questionnaire; CMS, Certainty about Mental States; IC, Interest and Curiosity in Mental states.

Table 3 also shows the descriptive statistics for the separate categories of mind‐related comments. Cultural differences in the separate categories of appropriate mind‐related comments were investigated using MANCOVA, with the separate categories added as dependent variables, nationality added as a fixed variable, and infant age, maternal education, HADS scores, the PSI‐SF subscale scores (parenting distress, parent–child dysfunctional interaction, and difficult child), and Lab‐TAB scores added as covariates. There was a main effect of nationality, *F*(4, 116) = 3.93, *p* = .005. Follow‐up tests showed a main effect of nationality on appropriate comments on (a) desires and preferences, *F*(1, 119) = 11.41, *p* < .001, *η* ^2^ = .085, (b) cognition, *F*(1, 119) = 4.95, *p* = .028, *η* ^2^ = .037, and (c) emotion, *F*(1, 119) = 8.38, *p* = .005, *η* ^2^ = .064; there was no main effect of nationality on intention, *F*(1, 119) = 0.52, *p* = .473, *η* ^2^ = .005. British mothers commented more frequently in appropriate ways on their infants’ desires and preferences than did Korean mothers, but Korean mothers made more appropriate mind‐related comments about their infants’ cognitive and emotional states compared with British mothers.

Cultural differences in the separate categories of non‐attuned mind‐related comments were investigated using MANCOVA as described above. There was a main effect of nationality, *F*(5, 115) = 4.75, *p* = .001. Follow‐up tests showed a main effect of nationality on non‐attuned comments on (a) desires and preferences, *F*(1, 119) = 6.10, *p* = .015, *η* ^2^ = .041, (b) emotion, *F*(1, 119) = 4.39, *p* = .038, *η* ^2^ = .035, and (c) physical states, *F*(1, 119) = 12.78, *p* = .001, *η* ^2^ = .092; there was no main effect of nationality on cognition, *F*(1, 119) = 2.47, *p* = .119, *η* ^2^ = .019, or intention, *F*(1, 119) = 0.99, *p* = .323, *η* ^2^ = .009. British mothers commented more frequently in non‐attuned ways on their infants’ desires and preferences than did Korean mothers, but Korean mothers made more non‐attuned mind‐related comments about their infants’ emotional and physical states compared with British mothers (see Table 3 for descriptive statistics).

The PRFQ data are shown in Table 3 as a function of nationality. Cultural differences in PRFQ were investigated using MANCOVA, with scores for *Certainty about mental states* and *Interest and curiosity about mental states* added as dependent variables, nationality added as a fixed variable, and infant age, maternal education, HADS scores, the PSI‐SF subscale scores (parenting distress, parent–child dysfunctional interaction, and difficult child), and Lab‐TAB scores added as covariates. The correlations between the variables are shown in Table 2. There was a main effect of nationality, *F*(2, 118) = 15.10, *p* < .001. Follow‐up tests showed a main effect of nationality on *Certainty about mental states*, *F*(1, 119) = 26.70, *p* < .001, *η* ^2^ = .135, but not *Interest and curiosity in mental states*, *F*(1, 119) = 0.61, *p* = .435, *η* ^2^ = .004. Korean mothers scored more highly than British mothers for *Certainty about mental states*.

### 3.3 Relations between parenting practices and certainty about mental states in British and Korean mothers

Our hypothesis related to whether mothers’ reported certainty about their infants’ mental states was related to more positive parenting, and whether any such relation was specific to Korean mothers. This hypothesis was investigated using self‐reported parenting quality and an observational measure of maternal intrusiveness. Self‐reported and observed parenting measures were unrelated in both Korean (*r*s .04 and −.05 for correlations between observed intrusiveness and self‐reported positive and negative parenting, respectively) and British (*r*s −.05 and .14 for correlations between observed intrusiveness and self‐reported positive and negative parenting, respectively) mothers. Scores for *Certainty about mental states* were (a) positively correlated with self‐reported positive parenting in Korean mothers, *r*(64) = .42, *p* < .001, and British mothers, *r*(61) = .41, *p* < .001, and (b) negatively correlated with self‐reported negative parenting in Korean mothers, *r*(64) = −.46, *p* < .001, and British mothers, *r*(61) = −.39, *p* = .002. In contrast, observed maternal intrusiveness was not related to *Certainty about mental states* in either Korean mothers, *r*(64) = −.13, *p* = .306, or British mothers, *r*(61) = −.19, *p* = .131 (Note that our aim was to test the specific relation between parenting and *Certainty about mental states*, but the correlations between the parenting variables and *Interest and curiosity in mental states* are reported in Table 2).

### 3.4 Relations between maternal mind‐mindedness and PRFQ dimensions in British and Korean mothers

Finally, relations between the two constructs of parental mentalization were explored. Correlations between appropriate and non‐attuned mind‐related comments and the PRF subscales were run separately for the British and Korean mothers. As shown in Table 4, there were no relations between the mind‐mindedness and PRF variables in either British or Korean mothers, with negligible to small effects for all correlations. Given that effect sizes in this range were predicted, additional Bayesian analyses were conducted to verify the null findings (see Table 4). We set the Bayes factor to BF_01_ to indicate the strength of evidence in favor of the null hypothesis. The Bayes factor indicates the degree to which the data are more likely to occur under the null hypothesis than the alternative hypothesis, with the value being interpreted with reference to specific conventions (Schmalz et al., 2023). For the UK sample, the Bayesian analyses indicate strong support for the null hypothesis for the correlation between appropriate mind‐related comments and *Certainty about mental states*, and moderate support for the null hypothesis for the correlation between (a) appropriate comments and *Interest and curiosity in mental states*, (b) non‐attuned comments and *Certainty about mental states*, and (c) non‐attuned comments and *Interest and curiosity in mental states*. For the Korean sample, the Bayesian analyses indicate moderate support for the null hypothesis for the correlation between (a) appropriate mind‐related comments and *Certainty about mental states*, (b) appropriate comments and *Interest and curiosity in mental states*, and (c) non‐attuned comments and *Certainty about mental states*, and anecdotal support for the null hypothesis for the correlation between non‐attuned comments and *Interest and curiosity in mental states*. Note that the correlations between *Interest and curiosity in mental states* and non‐attuned mind‐related comments are positive in the Korean sample.

**TABLE 4:** Associations between maternal mind‐mindedness and the PRFQ subscales.

		Appropriate mind‐related comments		Non‐attuned mind‐related comments	
		Pearson's *r*	Bayes factor	Pearson's *r*	Bayes factor
United Kingdom	CMS	.004	10.01	−.03	9.85
	IC	.06	8.94	.11	6.81
South Korea	CMS	.14	5.68	.18	3.82
	IC	.18	3.72	.20	2.88

CMS, Certainty about mental states; IC, Interest and curiosity in mental states; PRFQ, Parental Reflective Functioning Questionnaire.

## 4 DISCUSSION

The present study expands our understanding of cultural differences in parental mentalization. We hypothesized that the focus on *oneness* in Korean parenting would make Korean mothers more likely than their British counterparts to comment in both appropriate and non‐attuned ways on their infants’ internal states. Contrary to our hypothesis, no differences in either index of mind‐mindedness were observed across the two cultures. These findings contrast with those in the extant literature indicating that mothers from other Asian cultures are less mind‐minded than their Western counterparts (Dai et al., 2019; Fujita & Hughes, 2020; Hughes et al., 2018). As discussed in Section 1, oneness is unique to Korean parenting, and the lower levels of mind‐mindedness observed in Chinese and Japanese mothers compared with their British and Australian counterparts may therefore not provide strong grounds for expecting similar differences between Korean and Western mothers. Our findings thus highlight the need for caution in assuming equivalence in parenting across collectivistic Asian cultures and making a false dichotomy between “Western mind” versus “Eastern mind” (Harkness et al., 2000).

It is important to consider whether the observed lack of difference in mind‐mindedness between British and Korean mothers arose because the data from our British sample are unusual and therefore mask genuine cross‐cultural differences. In fact, the scores for appropriate and non‐attuned mind‐related comments of the British mothers in our study are very similar to those reported for other British samples (e.g., Larkin et al., 2019; Meins et al., 2012), and also to those of Dai et al.’s (2019) Australian sample. The lack of group difference in the present study thus does not appear to be because our sample of British mothers was less mind‐minded than other British samples reported in the literature.

Our hypothesized cultural differences were, however, observed in relation to the type of internal states on which Korean and British mothers commented. British mothers commented more than their Korean counterparts on their infants’ desires and preferences, whereas Korean mothers focused more on their infants’ cognitions and emotions. Korean mothers were also more likely than British mothers to make non‐attuned comments about their infants’ physical states. These findings likely reflect British mothers’ parenting goals of facilitating their infants’ individuality and agency, in line with their individualistic cultural context. Indeed, during the free play sessions, British mothers often started their play by asking about their infant's preference for different toys (e.g., “What would you like to play with?”), while most Korean mothers started by asking about an object label (e.g., “What is this?”). Korean mothers’ observed emphasis on cognition, emotion, and physical states compared with their British counterparts appears in line with their parenting goal of establishing relational closeness with their infants, and may thus represent a manifestation of oneness. Together with other findings in the extant literature, our results suggest that maternal mind‐mindedness may be universal, but that specific cultural contexts result in differences in the emphases placed on certain internal states. This is in line with Lillard's (1998) proposal that the specific features of mental states that are ascribed—rather than the overall propensity to talk about the mental states of others—differ depending on how the particular culture comprehends the concept of mind and its development.

Interestingly, these differences in the types of internal state emphasized in the two cultural contexts did not necessarily result in greater accuracy in interpreting such internal states. British mothers scored more highly for both appropriate and non‐attuned comments on desires and preferences than did Korean mothers, while Korean mothers scored more highly for both appropriate and non‐attuned comments relating to their infants’ emotions. These findings raise the intriguing possibility that a cultural focus on the importance of certain internal states may result in caregivers overinterpreting those states emphasized within their particular cultural context. For example, British individualistic culture may mean that caregivers interpret subtle cues as indicating that the infant wants a particular toy when in fact the infant may not evidence such a desire. Similarly, the Korean focus on oneness may increase the likelihood that mothers will attribute emotional or physical states to their infants in the absence of any behavioral cue. Although there is an established literature on the developmental outcomes of mind‐mindedness, with appropriate mind‐related comments predicting positive aspects of development and non‐attuned comments predicting less adaptive aspects of development (e.g., Meins et al., 2012, 2013), research has not yet charted how such predictive relations may be moderated by the types of internal state on which caregivers comment. Given the cultural differences with regard to the content of internal state speech observed in the present study, future research should consider differentiating not only between appropriate and non‐attuned mind‐related comments but also different categories of internal states. These more fine‐grained analyses of the content of caregivers’ mind‐related comments may prove important in understanding how mind‐mindedness predicts children's development across cultures. Future research could explore this issue cross‐culturally in relation to established correlates of early mind‐mindedness, such as attachment security and children's mentalizing abilities.

Turning to differences in PRF, the results supported the hypothesized higher scores for *Certainty about mental states* in Korean mothers compared to their British counterparts. In contrast, the two groups did not differ with respect to *Interest and curiosity in mental states*. Considering that the Korean parenting context emphasizes the sense of oneness between mother and infant, it is not surprising that Korean mothers are more likely than British mothers to consider themselves to know precisely what their infants are thinking and feeling. The *Certainty about mental states* and *Interest and curiosity in mental states* subscales had acceptable internal reliability in both cultural groups, suggesting that these aspects of PRF generalize across British and Korean cultures. However, the *Pre‐mentalizing modes* subscale was found to have low internal reliability in the Korean sample of mothers. Lee et al. (2021) reported that statements indicative of pre‐mentalizing modes did not load onto a single factor for Korean parents. This aspect of PRF may thus not show cross‐cultural generalizability. Interestingly, Arkle et al. (2023) assessed PRF longitudinally using the PRFQ in a British sample and reported low internal reliability in the *Pre‐mentalizing modes* subscale at both testing phases. These findings suggest that high levels of variance in this subscale may not apply only to Korean parents. Variability in the internal reliability of *Pre‐mentalizing modes* may be due to the fact that this subscale focuses on diverse aspects of parenting, including a range of ways in which caregivers may attribute malicious intent to the child (“My child sometimes gets sick to keep me from doing what I want to do”), lack of engagement with the child's mental world (“Often, my child's behavior is too confusing to bother figuring out”), and uncertainty about being loved by the child (“The only time I'm certain my child loves me is when he or she is smiling at me”). Future research should consider developing more culturally appropriate and sensitive measures of this aspect of parental mentalization, and further investigate whether the *Pre‐mentalizing modes* subscale comprises separate dimensions of PRF.

We also aimed to investigate whether certainty about mental states related to the quality of parenting, and whether any such relation was specific to Korean mothers. Higher certainty about mental states was positively correlated with self‐reported parenting quality in both Korean and British mothers. These findings are in line with those of Lee et al. (2021), who found that Korean mothers’ reported optimal parenting style was positively related to scores on the *Certainty about mental states* subscale. However, our findings indicate that this relation generalizes to British mothers, and thus appears not to result from having precise insight into the infant's internal states being regarded as ideal for Korean mothers. Regardless of the cultural parenting context, mothers who perceive themselves to have a positive parenting style showed higher levels of certainty about what their infants were thinking and feeling. In contrast, certainty about mental states was unrelated to observed parenting quality in both cultural groups. Greater certainty did not therefore translate into more sensitive parenting. The fact that the association with certainty about mental states was seen only for the self‐report measure of parenting suggests that, across cultures, caregivers who are more certain about their infants’ mental states may not be accurate judges of their parenting abilities; rather, they may demonstrate overconfidence in their ability to parent effectively and sensitively. Of course, shared method variance between the self‐report parenting and PRFQ measures may also explain the observed pattern of findings.

The present study was the first to investigate relations between the two parental mentalization constructs using a cross‐cultural design. In line with our hypotheses, no associations were observed between any of the indices of mind‐mindedness and PRF in either cultural group. These findings replicate the null results reported for relations between mind‐mindedness and PRF in previous research (Dollberg, 2022; Krink & Ramsauer, 2021), with effect sizes for the present study in line with the negligible to small effects previously reported. We used Bayesian analyses to confirm the findings, with these analyses indicating moderate to strong support for the null hypothesis for all relations apart from that between non‐attuned comments and interest and curiosity in mental states in Korean mothers, for which there was only anecdotal support for the null hypothesis. However, this correlation between non‐attuned comments and interest and curiosity in mental states was positive, indicating that mothers’ tendency to misinterpret their infants’ thoughts and feelings during infant–mother interaction was associated with higher self‐reported interest and curiosity in their infants’ internal states. These findings present robust evidence that mind‐mindedness and the PRFQ dimensions are unrelated.

There is thus a growing body of research indicating that the caregiver's tendency to reflect on the infant's mind in the context of an interview or questionnaire does not mean that caregivers will translate this reflection into their actual interactions with their infants, and consequently demonstrate higher levels of mind‐mindedness. These findings are relevant to our understanding of the umbrella construct of parental mentalization. The fact that a number of studies have reported null findings for relations between mind‐mindedness and PRF highlights the need for caution in assuming that the two constructs assess the same facet of caregiving, and support conceptualizing parental mentalization as a multidimensional construct. Further null results may necessitate a rethinking of theoretical perspectives on caregivers’ more general tendency to engage with their children's mental worlds and result in a move away from grouping constructs such as mind‐mindedness and PRF under the label *parental mentalization*.

Our results should be interpreted in light of certain limitations. First, the *Pre‐mentalizing modes* subscale was found to have low reliability in Korean mothers, and scores for this subscale were not included in the analyses. The present study was therefore unable to compare differences in this aspect of PRF cross‐culturally or to investigate how scores on this subscale of PRF related to the mind‐mindedness indices. Second, as all of the participants in the present study were mothers, with most from educated, middle‐class backgrounds, it cannot be assumed that our findings will generalize to other caregivers or populations. Lastly, our findings cannot speak to whether the observed cultural differences in parental mentalization are stable over time or across parents of different age groups.

In future research, it would be interesting to investigate how these cultural differences in parental mentalization predict children's later development. Certainty about the mental states of one's infant is considered to indicate a lack of awareness of the opacity of mental states and is thus taken to indicate lower levels of PRF and less optimal parenting in Western cultures (Luyten et al., 2017). However, the same might not be true of cultures such as Korea. If such certainty is culturally appropriate, it may be associated with positive aspects of child development. While findings like this would appear counter‐intuitive when interpreted with reference to Western parenting ideals, the same does not hold from the perspective of Korean parenting ideals. On the other hand, although maternal mind‐mindedness is known to predict children's mentalizing ability in Western countries (e.g., Kirk et al., 2015; Laranjo et al., 2010; Lundy, 2013; Meins et al., 2003), little is known about the link between mind‐mindedness and the development of children's mentalizing skills in non‐Western countries. Sensitive future research on different populations and cultures can therefore enrich our understanding of the parenting role and the many ways in which parenting relates to children's development.

## CONFLICT OF INTEREST STATEMENT

The authors declare no conflicts of interest.

## Data Availability

The data that support the findings of this study are openly available in the Open Science Framework at https://osf.io/mpn95/.
